# CHARACTERIZATION OF FISH AND REPTILIAN PARVALBUMINS AS RELEVANT FOOD ALLERGENS

**DOI:** 10.51894/001c.123077

**Published:** 2024-08-30

**Authors:** Andrea O’Malley

**Affiliations:** 1 College of Osteopathic Medicine Michigan State University

## 74

### INTRODUCTION

Fish allergy affects up to 3% of the population, with the majority of fish-allergic individuals having IgE recognizing β-parvalbumins. It was shown that fish allergic individuals are at risk of allergic reaction when consuming meat from crocodiles. Additionally, several parvalbumins such as the β-parvalbumin from cod have been noted to oligomerize, which may have an impact on its allergenicity.

### OBJECTIVES

Comparative studies of fish and reptilian parvalbumins will help to understand the molecular basis of the cross-reactivity and can improve generation of avoidance guidelines for parvalbumin-allergic patients. Biochemical, structural, and immunological studies will be performed in pursuit of this goal.

### METHODS

Gad m 1.0201 (Atlantic cod), Cro p 1.0101 and Cro p 2.0101 (saltwater crocodile), human α-parvalbumin, and α-parvalbumins from *Raja clavata* and *Callorhinchus millii* were recombinantly produced in *E. coli*. The produced proteins were used for structural, stability and antibody binding studies. Differential scanning fluorimetry was used for assessment of thermal stability, X-ray crystallography and NMR were used for structural characterization, and ELISA and immunoblotting were used for antibody binding.

### RESULTS

The studied parvalbumins displayed remarkable thermal stability, which is important from the perspective of food processing, and it was shown that calcium cations are necessary for protein stability. Five novel structures were determined via x-ray crystallography. They not only provided a detailed picture of the parvalbumins’ structures, but they also revealed the existence of a domain-swapped Gad m 1 dimer. Cross-reactivity between fish and reptilian parvalbumins was demonstrated.

### CONCLUSIONS

Further analysis of the structural and biochemical properties of α- and β-parvalbumin will allow for better understanding of the allergic response to seafood parvalbumin as well as the protein’s capacity for oligomerization.

